# Supplementary material for: Synthesis, DFT Calculations, Antiproliferative, Bactericidal Activity and Molecular Docking of Novel Mixed-Ligand Salen/8-Hydroxyquinoline Metal Complexes
Source: Molecules. 2021 Aug 4;26(16):4725. doi: 10.3390/molecules26164725 (PMC8401699; doi:10.3390/molecules26164725)
Supplement: Supplementary file 1 [file molecules-26-04725-s001.zip › molecules-1283722-supplementary.pdf]

Table (S1): Results of antibacterial bioassay of Salen ligand, metal salt and its mixed 8-hydroxy quinoline with Co, Ni, Cd, Al, La complexes against different strains of bacteria and fungi

Compound		Mic of Bacteria mg ml^{-1}			
		S. aureus (+ve)	B. subtilis (+ve)	E. coli (−ve)	P. vulgaris(−ve)
S		14	13	12	15
Q		16	14	13	16
Gentamycin		24	26	30	25
CoSQ		18	15	13	13
NiSQ		20	16	14	15
CdSQ		26	30	28	30
AlSQ		31	25	23	24
LaSQ		29	30	20	27
$CoCl_2.6H_2O$		15	10	9	11
$NiCl_2.6H_2O$		16	9	12	13
$CdCl_2.H_2O$		19	23	24	24
$AlCl_3.6H_2O$		25	18	17	19
$LaCl_3.7H_2O$		22	26	14	23
Ref. 43	CoSI	11	13	11	12
	NiSI	12	15	12	13
	CdSI	20	33	23	25
	AlSI	NA	NA	14	NA
	LaSI	12	13	NA	NA

Table (S2): Results of antifungal bioassay of Salen ligand, metal salt and its mixed 8-hydroxy quinoline with Co, Ni, Cd, Al, La complexes against different strains of bacteria and fungi

Compound		Mic of Fungi mg ml^{-1}	
		Candida albicans	Aspergillus flavus
S		0	0
Q		13	16
Ketoconazole		16	20
CoSQ		0	0
NiSQ		0	0
CdSQ		23	28
AlSQ		37	28
LaSQ		38	25
$CoCl_2.6H_2O$		0	0
$NiCl_2.6H_2O$		0	0
$CdCl_2.H_2O$		14	20
$AlCl_3.6H_2O$		29	20
$LaCl_3.7H_2O$		27	19
Ref. 43	CoSI	0	0
	NiSI	0	0
	CdSI	20	30
	AlSI	0	0
	LaSI	0	0

Table S3: Important optimized bond lengths (Å) and bond angles (°) of **[Co(S)(Q)(H_2O)]:**

Type of bond	Bond length(Å) Complex	Type of bond	Bond length(Å) S	Bond length(Å) Complex
Co-N1	1.908	Co-O3	-	2.162
Co-N2	2.272			
Co-N3	1.993	N1•••N2	3.788	2.709
Co-O4	2.113	N1•••O2	4.261	2.720
Co-O2	2.007	N2•••O2	7.003	3.045

Type of Angle	Angle (°) Complex	Type of Angle	Angle (°) Complex
N1-Co-N2	80.28	O3-Co-N1	91.58
N3-Co-O4	82.38	O3-Co-N2	93.53
N1-Co-O4	91.04	O3-Co-N3	90.01
N2-Co-N3	106.6	O3-Co-O4	79.26
O2-Co-N1	88.00	N1-Co-N3	172.8
O2-Co-N2	90.52	N2-Co-O4	168.6
O2-Co-N3	89.90	O2-Co-O3	175.7
O2-Co-O4	96.57	N1-N2-N3-O4	6.999*

*dihedral angle

Table (S4): Results of Important optimized bond lengths (Å) and bond angles (°) of [Ni(S)(Q)(H_2O)]

Type of bond	Bond length(Å) Complex	Type of bond	Bond length(Å) S	Bond length(Å) Complex
Ni-N1	2.151	Ni-O3	-	1.997
Ni-N2	2.249			
Ni-N3	2.288	N1•••N2	3.788	2.777
Ni-O4	2.071	N1•••O2	4.261	2.717
Ni-O2	1.860	N2•••O2	7.003	2.950

Type of Angle	Angle (°) Complex	Type of Angle	Angle (°) Complex
N1-Ni-N2	78.22	O3-Ni-N1	93.93
N3-Ni-O4	78.98	O3-Ni-N2	92.55
N1-Ni-O4	100.1	O3-Ni-N3	87.35
N2-Ni-N3	102.8	O3-Ni-O4	83.65
O2-Ni-N1	84.96	N1-Ni-N3	178.3
O2-Ni-N2	91.27	N2-Ni-O4	175.7
O2-Ni-N3	93.69	O2-Ni-O3	175.7
O2-Ni-O4	92.46	N1-N2-N3-O4	-3.917*

*dihedral angle

Table (S5): Results of Important optimized bond lengths (Å) and bond angles (°) of [Cd(S)(Q)(H_2O)]:

Type of bond	Bond length(Å) Complex	Type of bond	Bond length(Å) S	Complex
Cd-N1	2.227	Cd-O3	-	2.372
Cd-N2	2.409			
Cd-N3	2.312	N1•••N2	3.788	2.886
Cd-O4	2.219	N1•••O2	4.261	2.874
Cd-O2	2.231	N2•••O2	7.003	3.349
**Type of Angle**	**Angle (°) Complex**	**Type of Angle**		**Angle (°) Complex**
N1-Cd-N2	76.87	O3-Cd-N1		94.14
N3-Cd-O4	77.40	O3-Cd-N2		90.38
N1-Cd-O4	99.93	O3-Cd-N3		90.11
N2-Cd-N3	106.2	O3-Cd-O4		84.06
O2-Cd-N1	80.28	N1-Cd-N3		174.7
O2-Cd-N2	92.34	N2-Cd-O4		173.4
O2-Cd-N3	95.24	O2-Cd-O3		173.1
O2-Cd-O4	92.80	N1-N2-N3-O4		-7.655*

*dihedral angle

Table (S6): Results of Important optimized bond lengths (Å) and bond angles (°) of $[Al(S)(Q)(H_2O)]^+$

Type of bond	Bond length(Å) Complex	Type of bond	Bond length(Å) S	Bond length(Å) Complex
Al- N1	1.974	Al- O3	-	2.052
Al- N2	2.122			
Al- N3	2.065	N1•••N2	3.788	2.674
Al- O4	1.891	N1•••O2	4.261	2.673
Al- O2	1.869	N2•••O2	7.003	2.872

Type of Angle	Angle (°) Complex	Type of Angle	Angle (°) Complex
N1-Al- N2	81.42	O3-Al- N1	90.01
N3-Al- O4	83.86	O3-Al- N2	87.42
N1-Al- O4	95.59	O3-Al- N3	88.39
N2-Al- N3	-104.9	O3-Al- O4	82.89
O2-Al- N1	88.13	N1-Al- N3	178.4
O2-Al- N2	92.34	N2-Al- O4	169.9
O2-Al- N3	91.84	O2-Al- O3	178.1
O2-Al- O4	97.74	N1-N2-N3-O4	-5.790*

*dihedral angle

Table S7: Important optimized bond lengths (Å) and bond angles (°) of **[La(S)(Q)*Cl*]**

Type of bond	Bond length(Å) Complex	Type of bond	Bond length(Å) L	Complex
La-N1	2.534	La-O2	-	2.396
La-N2	2.704			
La-O3	2.402	N1- - - - -N2	3.788	2.991
La-N3	2.621	N1- - - - -O2	4.261	3.022
La-*Cl*	2.828	N2- - - - -O2	7.003	3.674
**Type of Angle**	**Angle (°) Complex**	**Type of Angle**	**Angle (°) Complex**	
N1-La- N2	69.55	*Cl*-La-N1	94.01	
N3-La-O3	69.70	*Cl*-La-N2	88.38	
N1-La-O3	107.2	*Cl*-La-N3	93.10	
N2-La-N3	113.5	*Cl*-La-O3	91.66	
O2-La-N1	75.55	N1-La-N3	172.3	
O2-La-N2	91.96	N2-La-O3	176.8	
O2-La-N3	97.13	O2-La-*Cl*	168.7	
O2-La-O3	87.36	N1-N2-N3-O3	-5.859*	

*dihedral angle

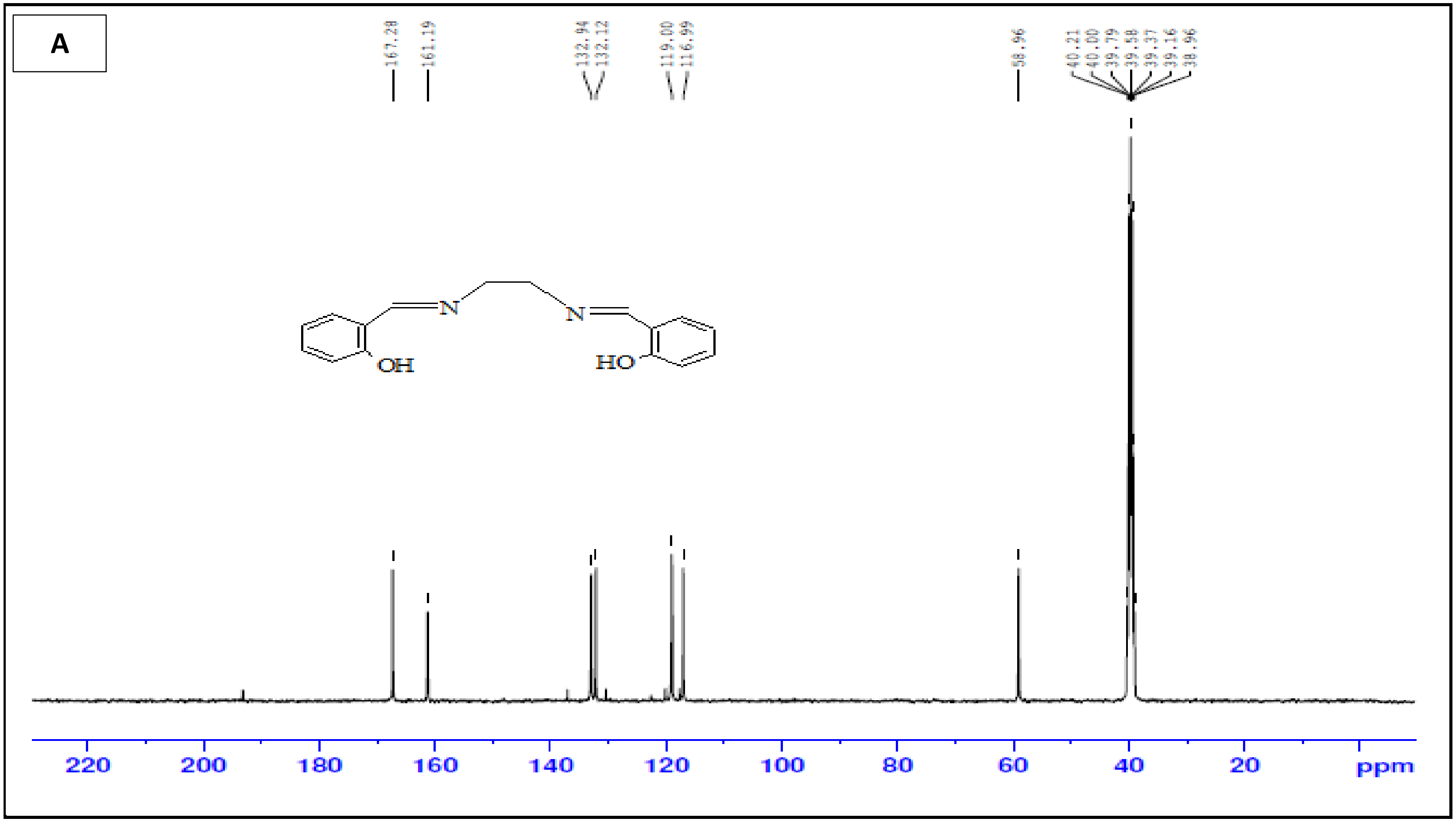
A
167.28
161.19
132.94
132.12
119.00
116.99
58.96
40.21
40.00
39.79
39.58
39.37
39.16
38.96
N
N
OH
HO
220
200
180
160
140
120
100
80
60
40
20
ppm

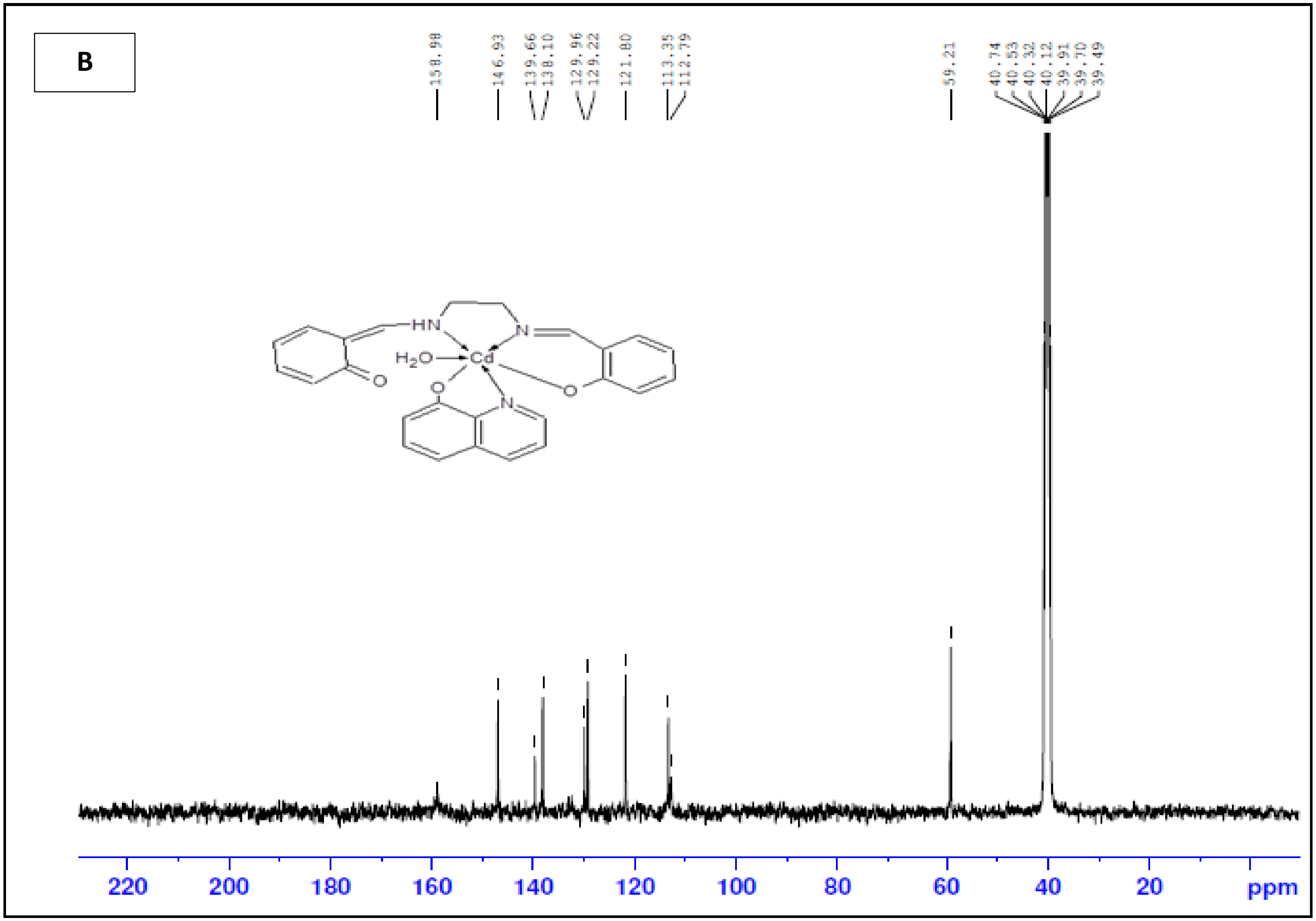
B
158.98
146.93
139.66
138.10
129.96
129.22
121.80
113.35
112.79
59.21
40.74
40.53
40.32
40.12
39.91
39.70
39.49
HN
N
H2O
Cd
O
O
O
N
220
200
180
160
140
120
100
80
60
40
20
ppm

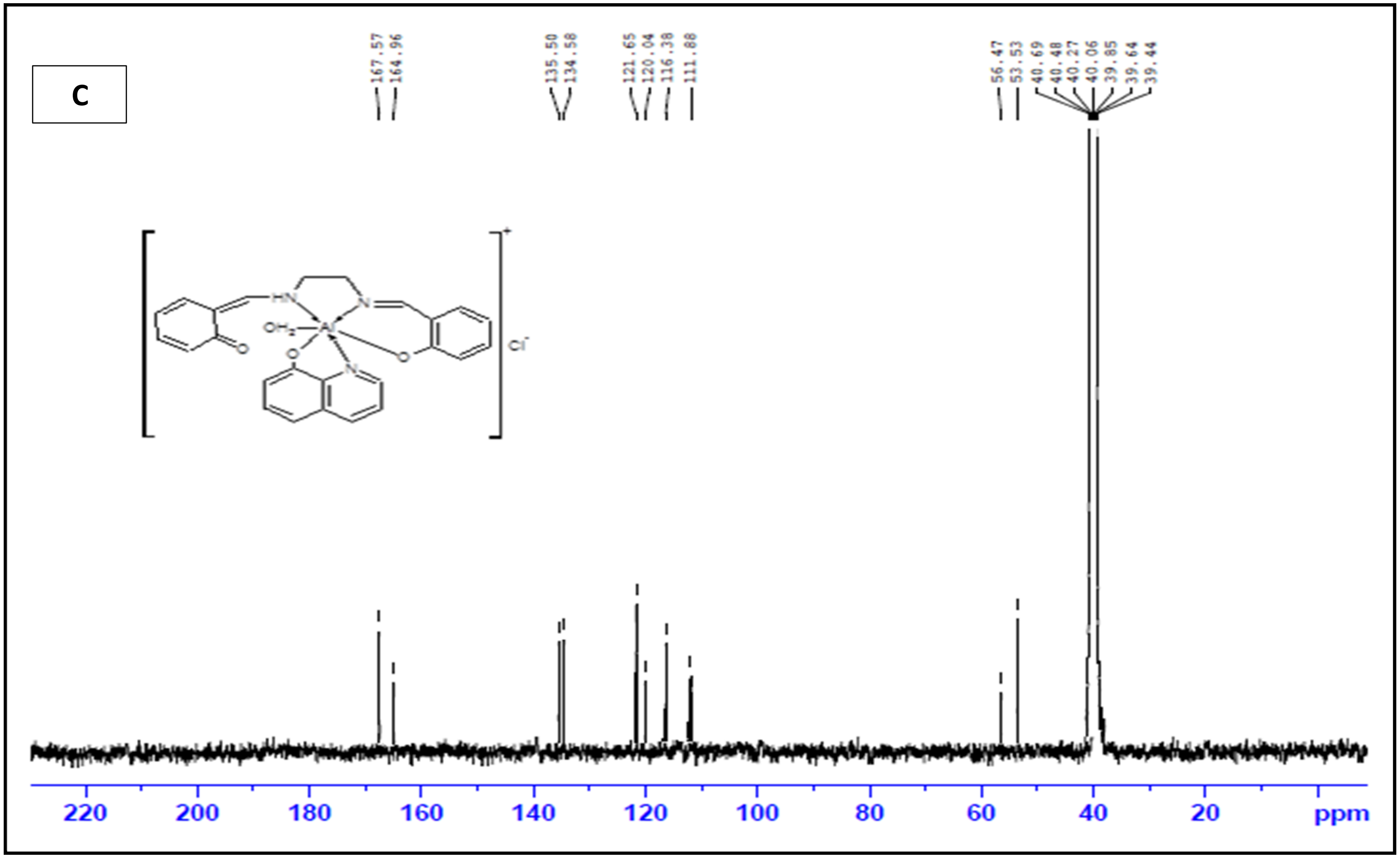

C
167.57
164.96
135.50
134.58
121.65
120.04
116.38
111.88
56.47
53.53
40.69
40.48
40.27
40.06
39.85
39.64
39.44
220
200
180
160
140
120
100
80
60
40
20
ppm

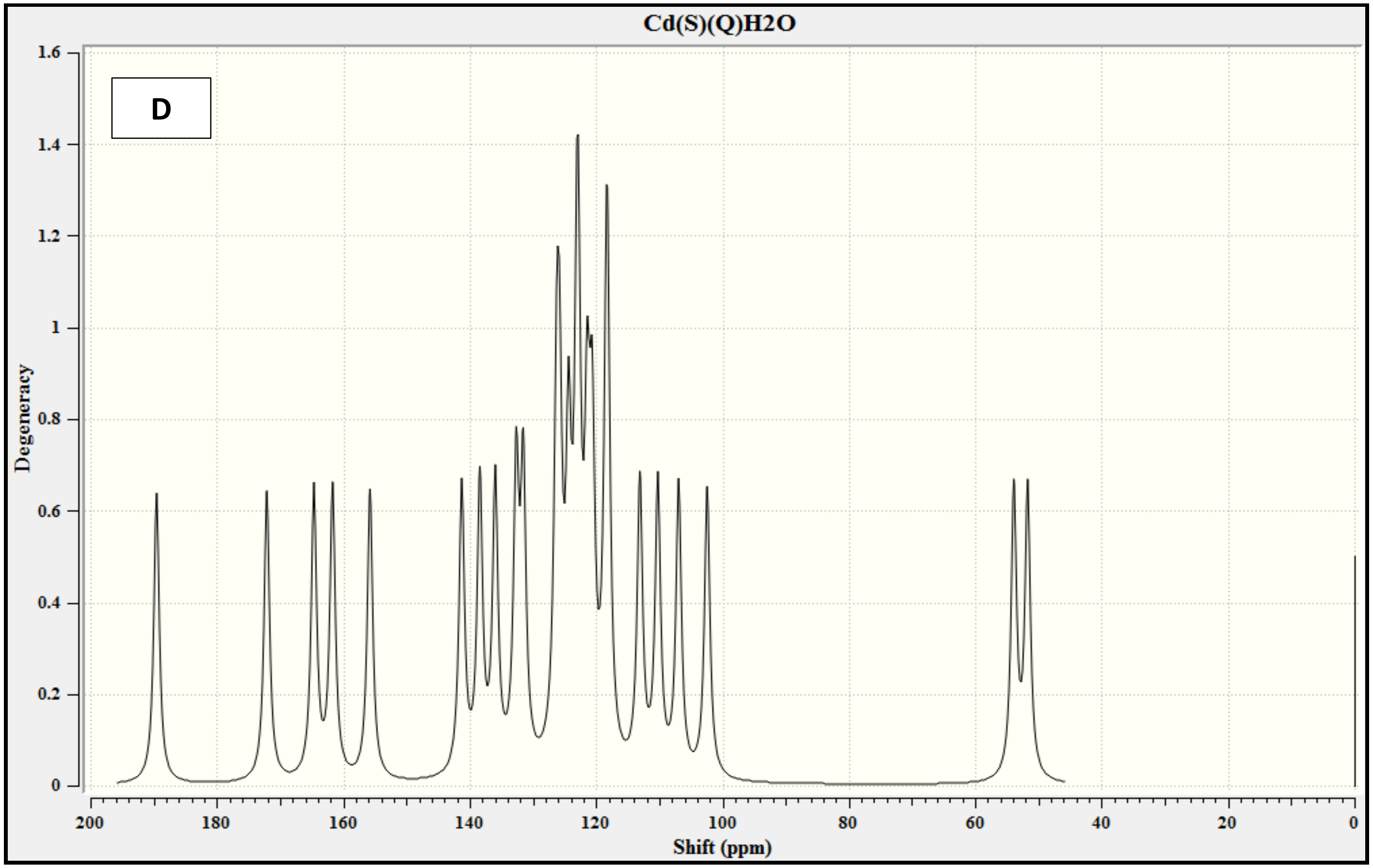
Cd(S)(Q)H2O
D
Degeneracy
1.6
1.4
1.2
1
0.8
0.6
0.4
0.2
0
200
180
160
140
120
100
80
60
40
20
0
Shift (ppm)

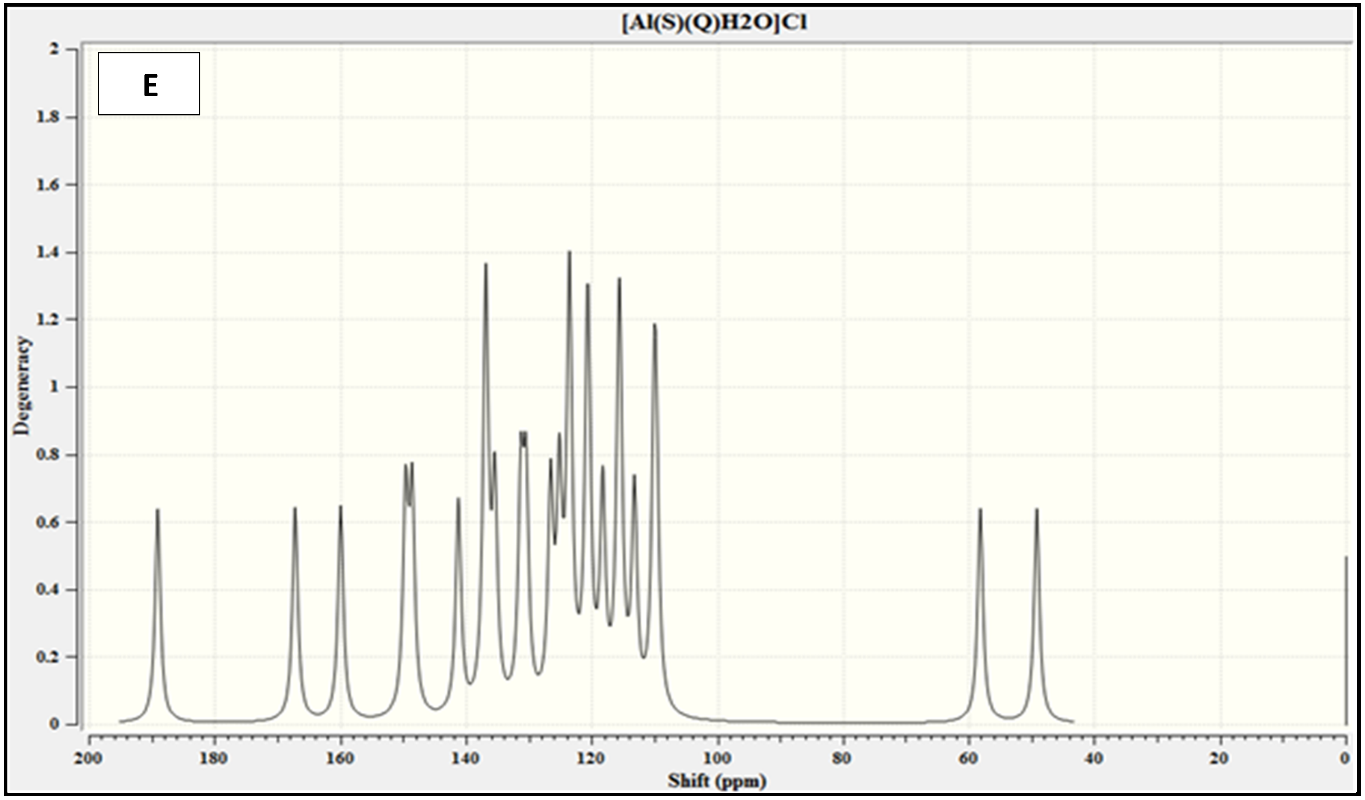

Figures (S1): ^{13}C NMR spectra of Salen ligand (A) and Cd(B and D), Al(C and E) mixed 8-hydroxy quinoline complexes

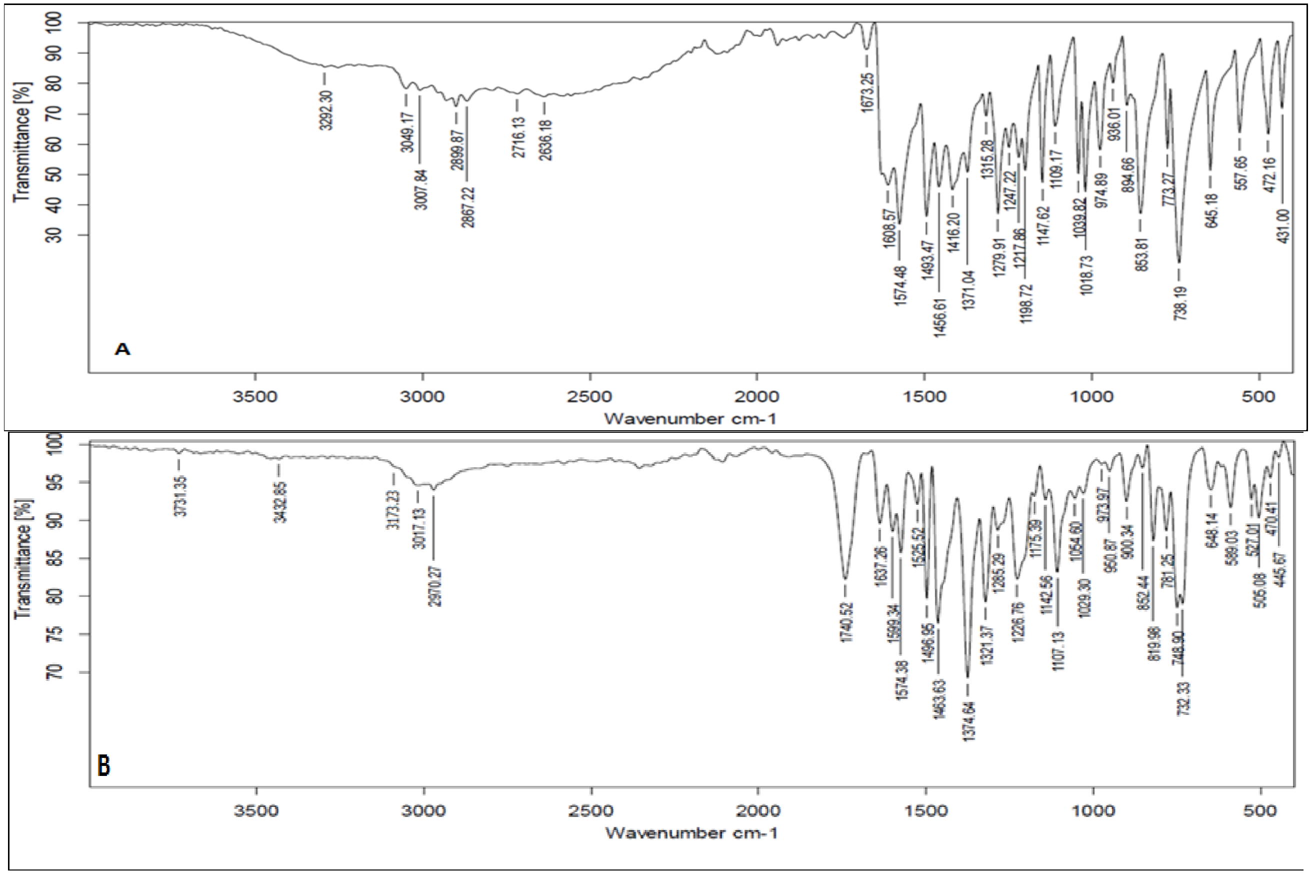
A
Transmittance [%]
Wavenumber cm-1
3292.30
3049.17
3007.84
2899.87
2867.22
2716.13
2636.18
1673.25
1608.57
1574.48
1493.47
1456.61
1416.20
1371.04
1315.28
1279.91
1247.22
1217.86
1198.72
1147.62
1109.17
1039.82
1018.73
974.89
936.01
894.66
853.81
773.27
738.19
645.18
557.65
472.16
431.00
B
3731.35
3432.85
3173.23
3017.13
2970.27
1740.52
1637.26
1599.34
1574.38
1525.52
1496.85
1463.63
1374.64
1321.37
1285.29
1226.76
1175.39
1142.56
1107.13
1054.60
1029.30
973.97
950.87
900.34
852.44
819.96
781.25
748.90
732.33
648.14
589.03
527.01
505.08
470.41
445.67

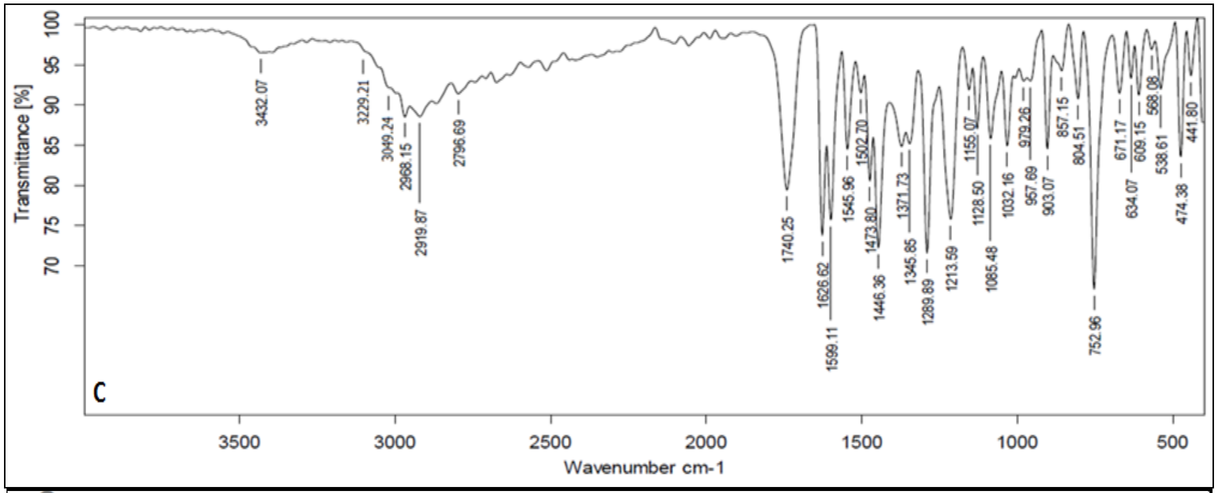
C
Transmittance [%]
100
95
90
85
80
75
70
3500
3000
2500
2000
1500
1000
500
Wavenumber cm-1
3432.07
3229.21
3049.24
2968.15
2919.87
2796.69
1740.25
1626.62
1599.11
1545.96
1502.70
1473.80
1446.36
1371.73
1345.85
1289.89
1213.59
1155.07
1128.50
1085.48
1032.16
979.26
957.69
903.07
857.15
804.51
752.96
671.17
634.07
609.15
568.08
538.61
474.38
441.80

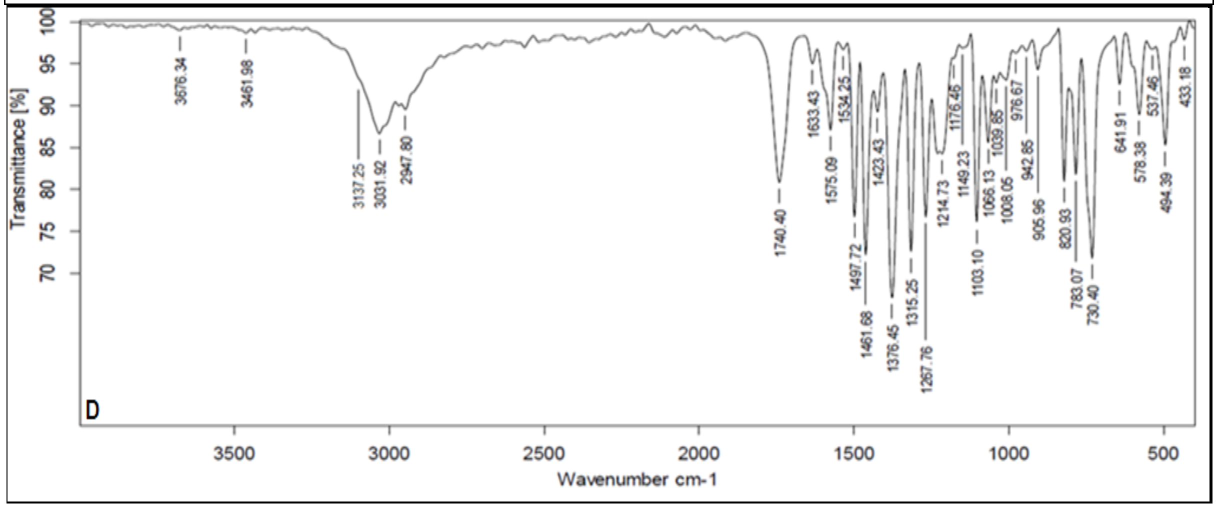
D
Transmittance [%]
100
95
90
85
80
75
70
3500
3000
2500
2000
1500
1000
500
Wavenumber cm-1
3676.34
3461.98
3137.25
3031.92
2947.80
1740.40
1633.43
1575.09
1534.25
1497.72
1461.68
1423.43
1376.45
1315.25
1267.76
1214.73
1176.46
1149.23
1103.10
1066.13
1039.85
1008.05
976.67
942.85
905.96
820.93
783.07
730.40
641.91
578.38
537.46
494.39
433.18

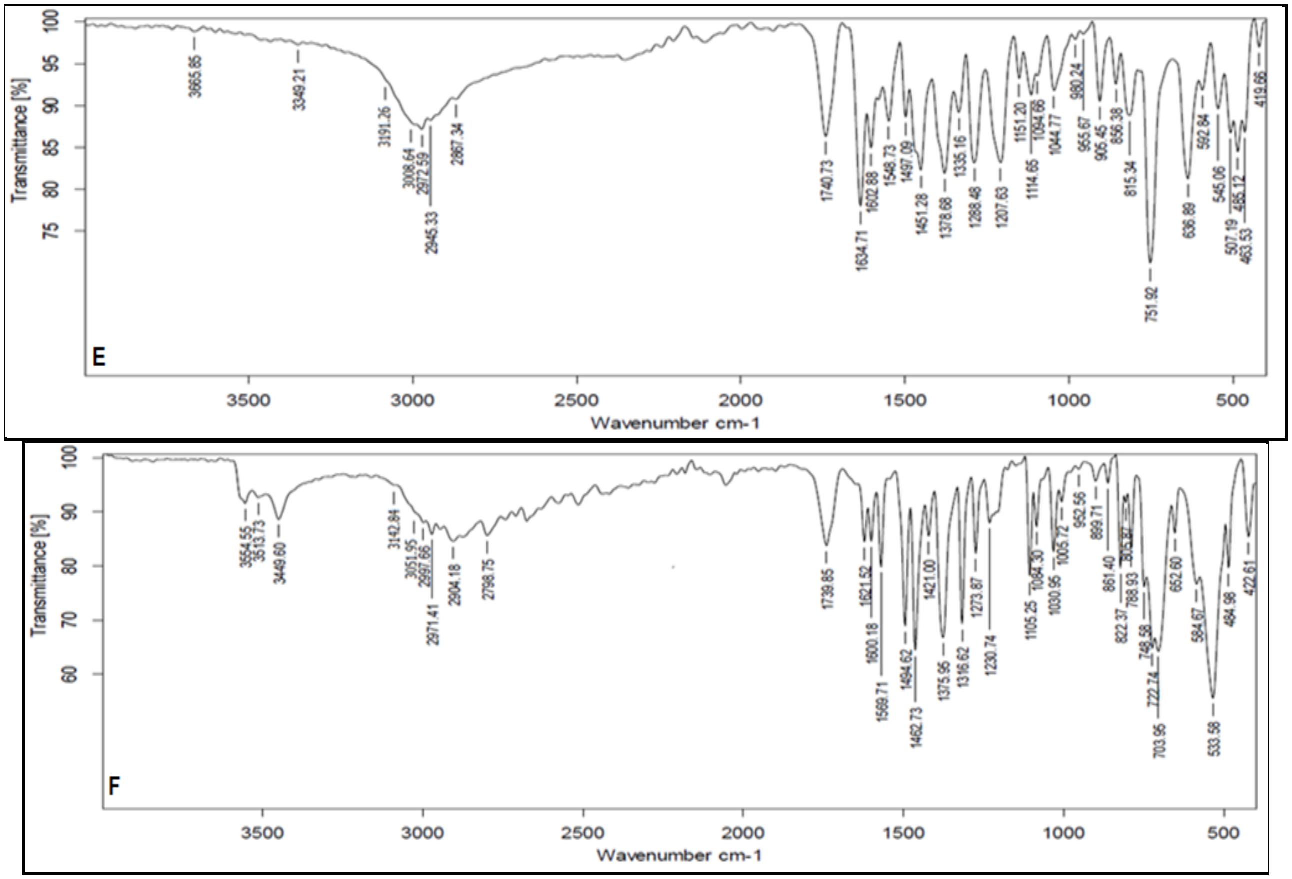

Figures (S2) : FT IR spectral of Salen Schiff base ligand (A) and its mixed 8-hydroxy quinoline (B)Co, (C)Ni, (D)Cd, (E)Al, (F)La complexes in 4000-400 cm^{-1}

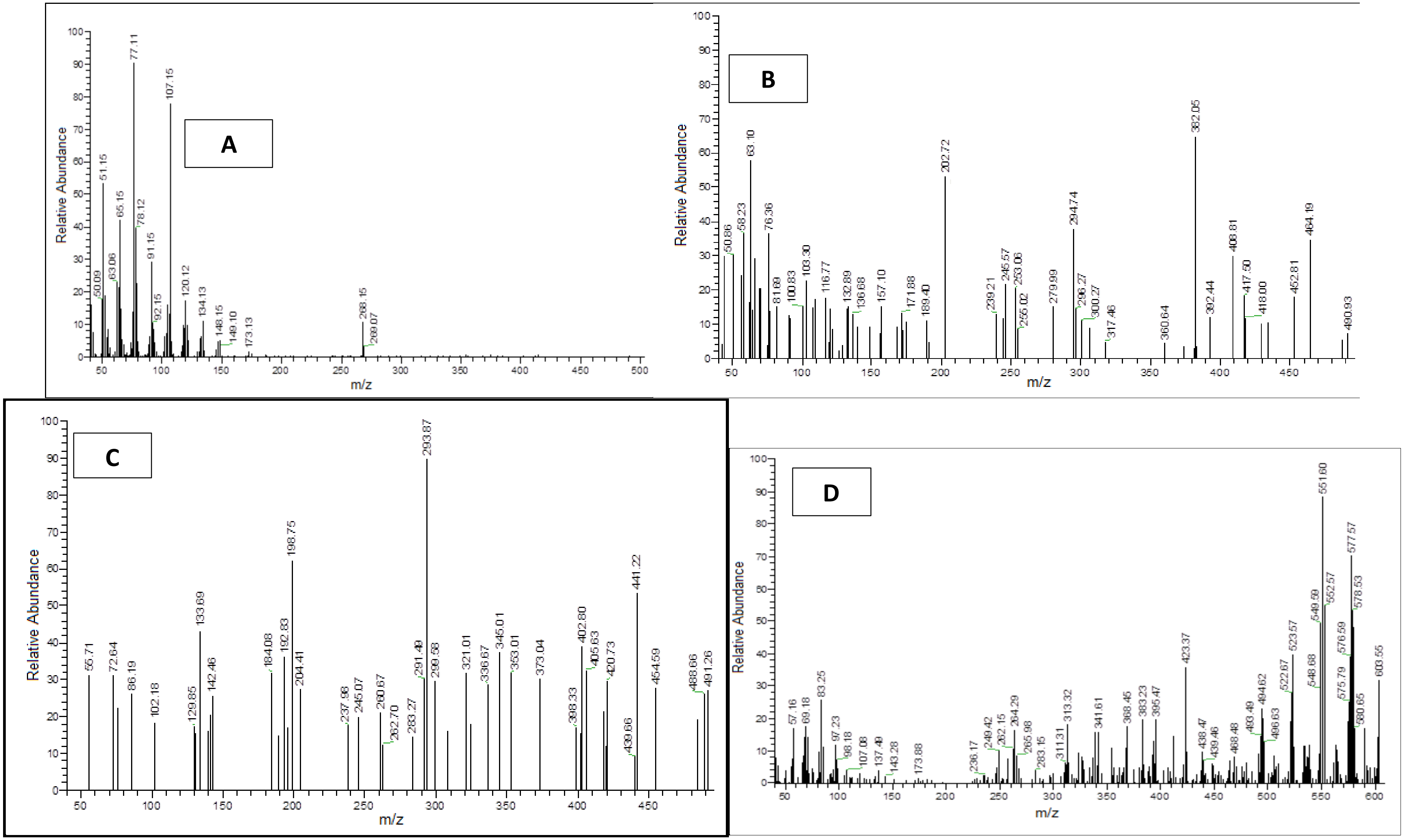

Figures (S3): The mass spectrum of Salen Schiff base ligand (A) and its mixed 8-hydroxy quinoline (B)CoSQ, (C)AlSQ, (D)LaSQ complexes

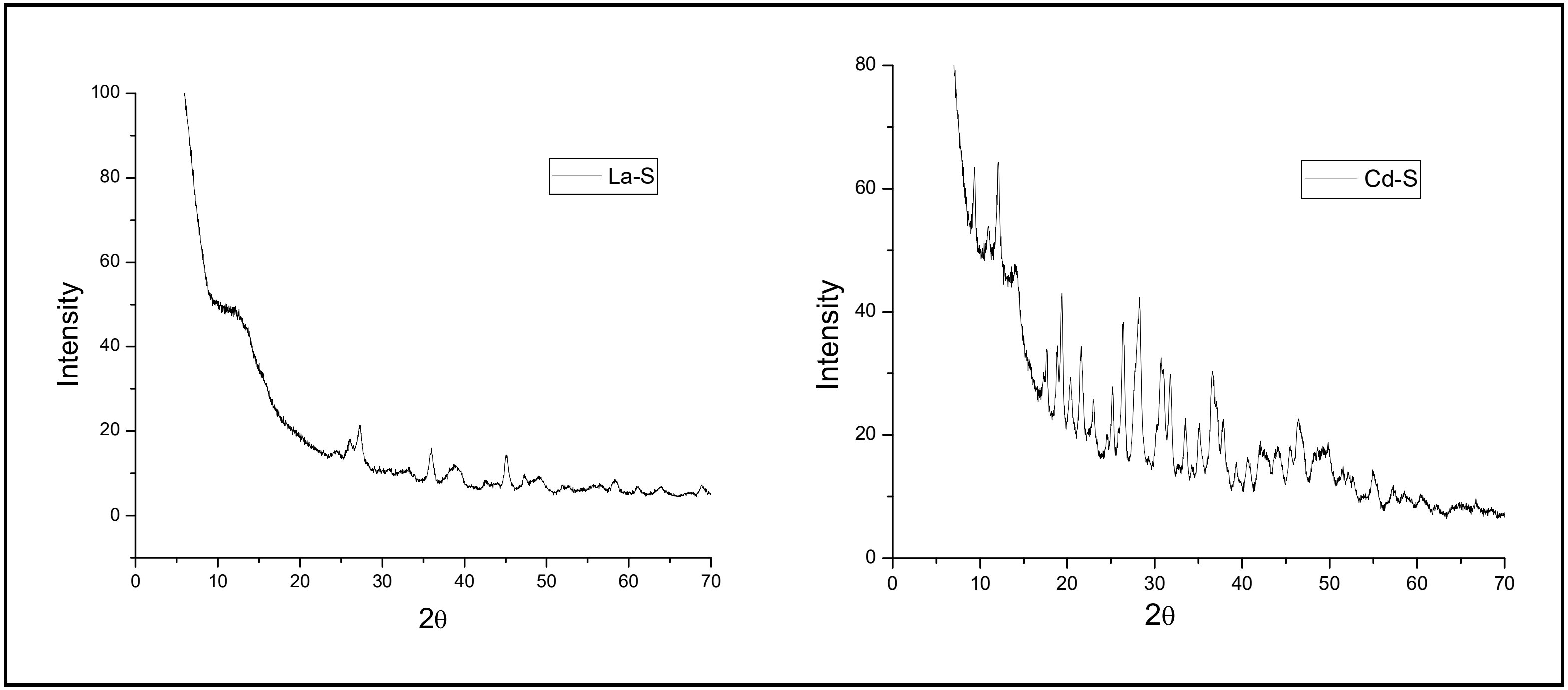

Figures (S4): PXRD powder pattern of Salen and its mixed CdSQ and LaSQ complexes. with 8-hydroxy quinolone

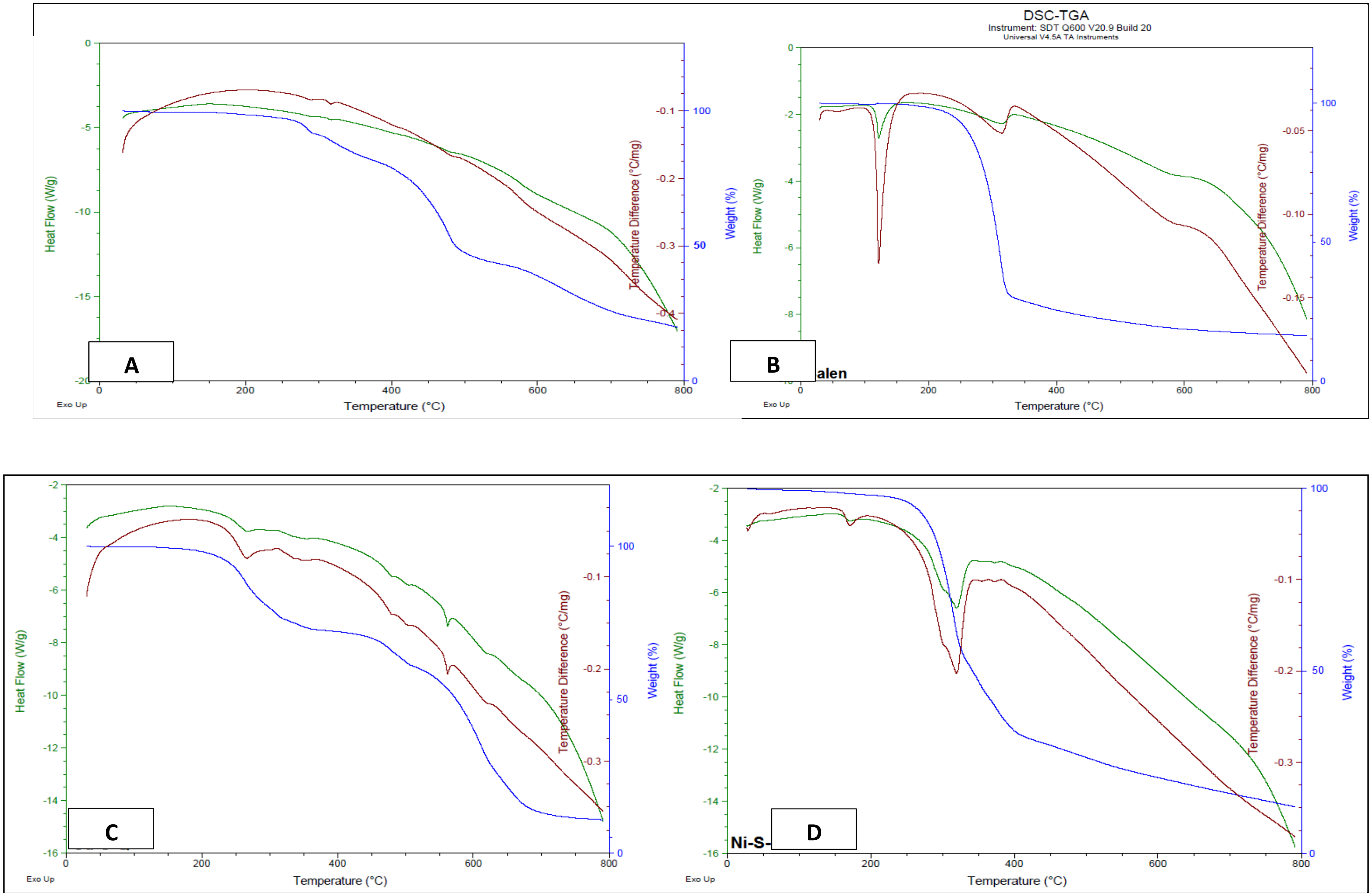
A
B
C
D
DSC-TGA
Instrument: SDT Q600 V20.9 Build 20
Universal V4.5A TA Instruments
alen
Ni-S-
Heat Flow (W/g)
Temperature Difference (°C/mg)
Weight (%)
Temperature (°C)
Exo Up

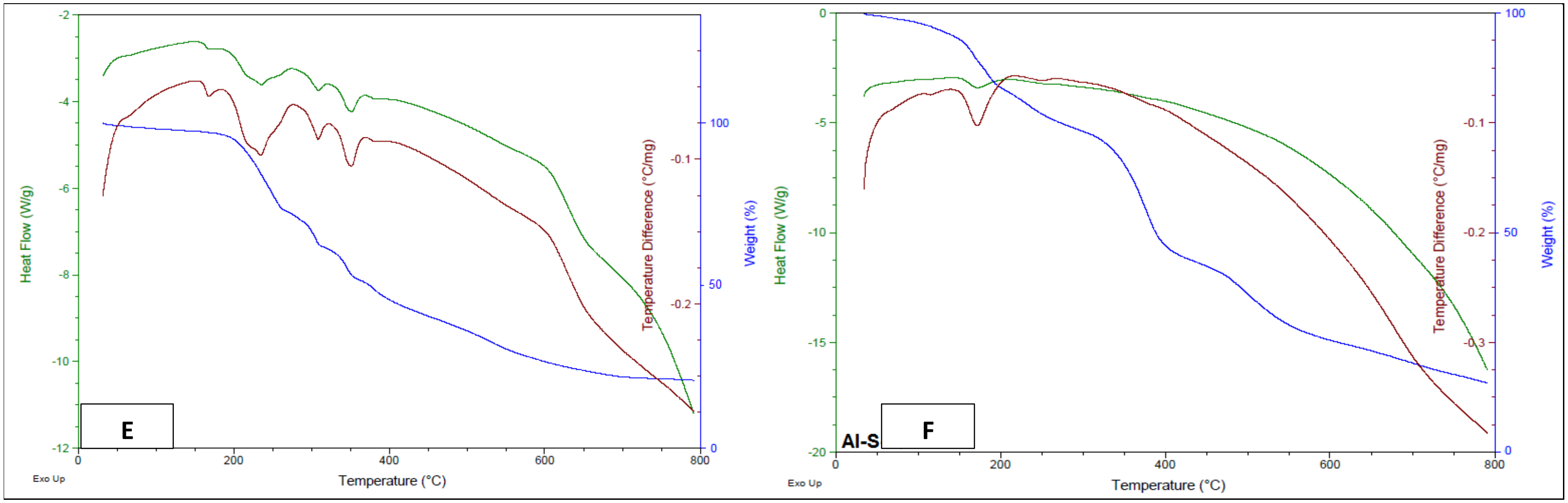

Figures (S5): Characteristic DSC-TGA curve of Salen ligand (A) and and its mixed 8-hydroxy quinoline (B)CoSQ, (C)NiSQ, (D)CdSQ, (E)AlSQ, (F)LaSQ complexes

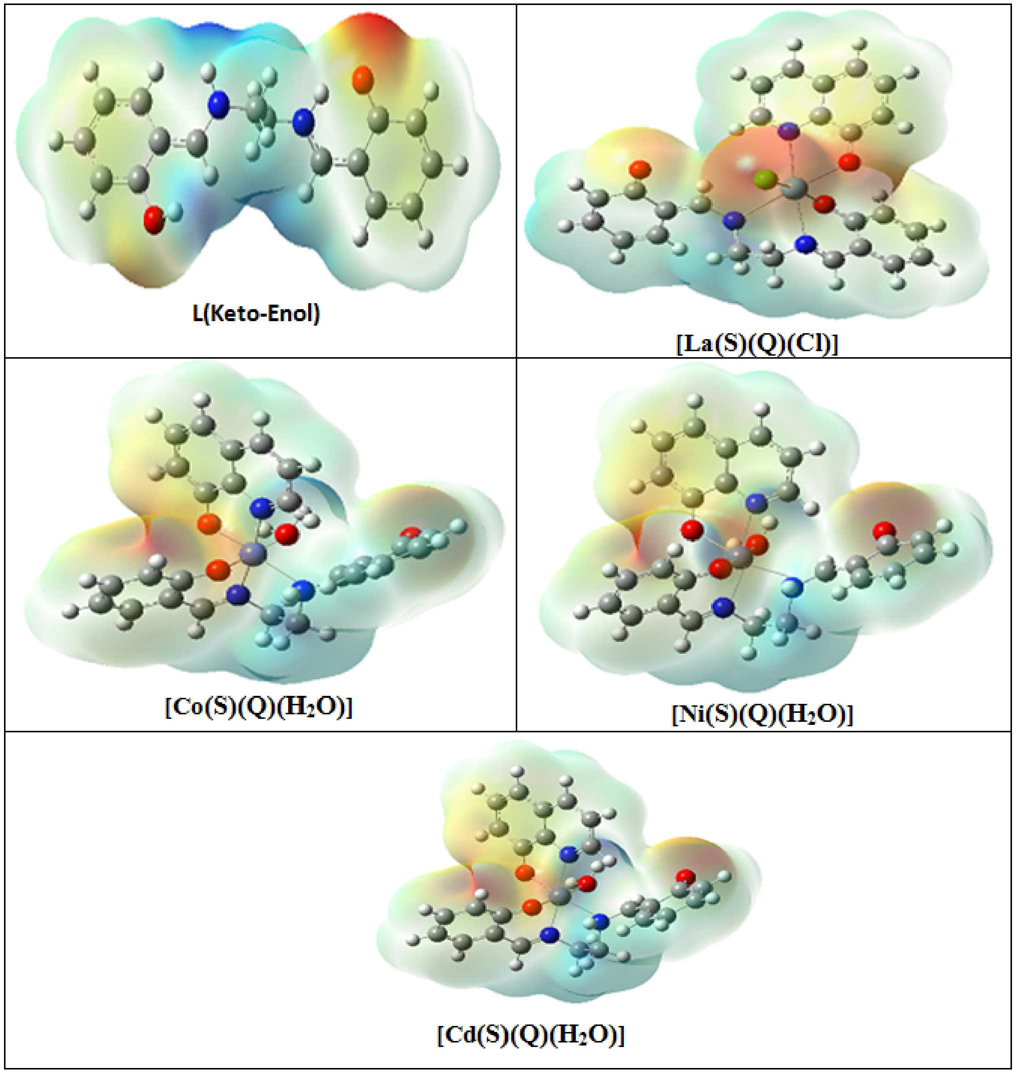

Figure S6: Molecular electrostatic potential (MEP) surface of ligand, L (Keto-Enol) and complexes [Co(S)(Q)(H_2O)], [Ni(S)(Q)(H_2O)], [Cd(S)(Q)(H_2O)], and [La(S)(Q) *Cl*] using B3LYP/LANL2DZ.

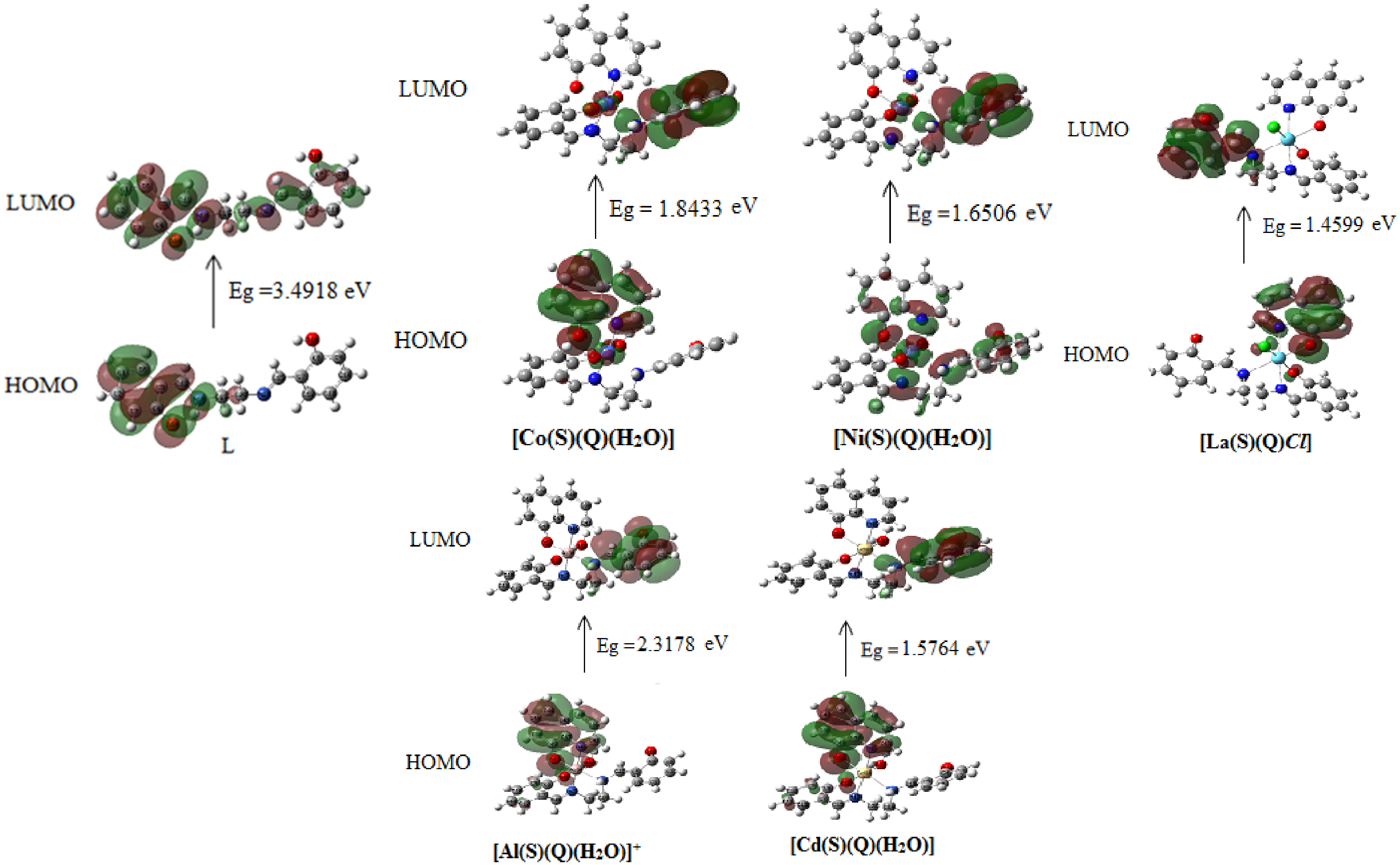

Figure S7: HOMO and LUMO charge density maps of ligand and complexes [Co(S)(Q)(H_2O)], [Ni(S)(Q)(H_2O)], [Cd(S)(Q)(H_2O)], [Al(S)(Q)(H_2O)]$^+$ and [La(S)(Q)(*Cl*)] using B3LYP/LANL2DZ

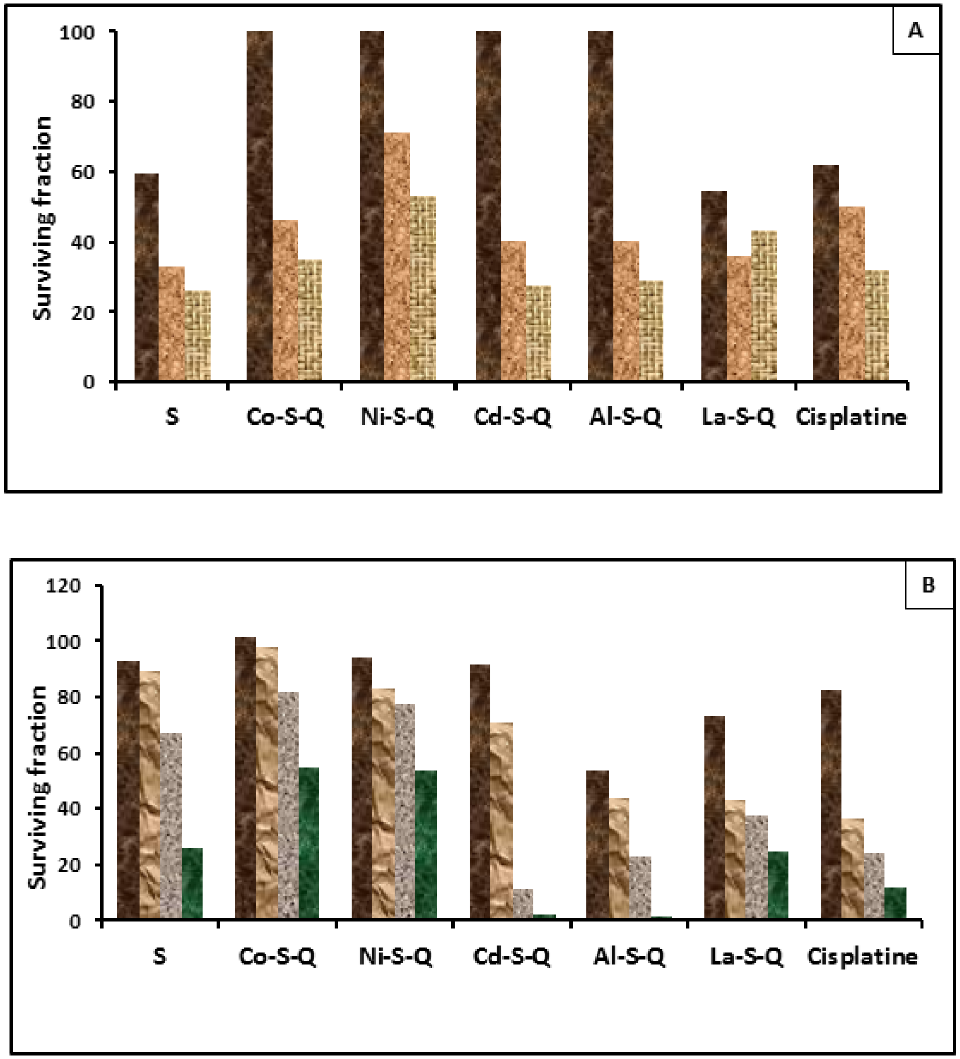

Figure(S8): Graph showing values of % viability of (A) Hep-G2 and (B)MDA-MB231 breast cell lines in different concentration for Salen and its Co(II), Ni(II), Cd(II), Al(III), La(III) mixed 8-hydroxy quinoline -Salen Complexes

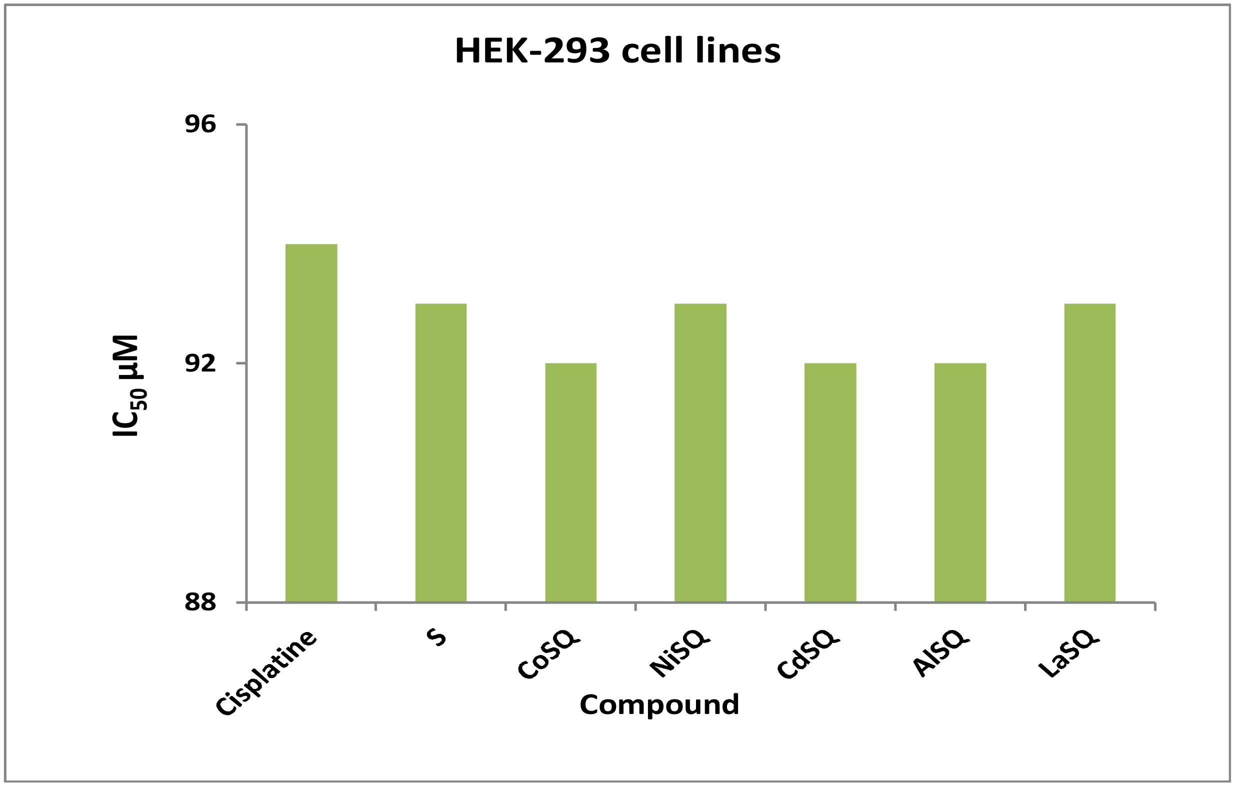

Figure S9: IC50 Values of Salen and its Cd(II), Al(III) and La(III) mixed 8-hydroxy quinoline complexes against HEK-293 cell lines compared to cisplatin.

$$[C_{25}H_{23}N_3O_4Ni]\tfrac{1}{2}H_2O \xrightarrow[-3/2H_2O]{25°C - 260°C} [C_{25}H_{21}N_3O_3Ni] \xrightarrow[-C_{11}H_{12}N_2O_2]{260°C - 320°C} [C_{14}H_9NONi] \xrightarrow[-C_8H_4NO]{320°C - 400°C}$$

$$[C_6H_5Ni] \xrightarrow[-C_6H_5]{400°C - 620°C} Ni$$

$$[C_{25}H_{23}N_3O_4Cd] \xrightarrow[-H_2O]{35°C - 200°C} [C_{25}H_{21}N_3O_3Cd] \xrightarrow[-C_5H_{10}NO_2]{200°C - 340°C} [C_{20}H_{11}N_2OCd] \xrightarrow[-CH_2O]{340°C - 465°C}$$

$$[C_{19}H_9N_2Cd] \xrightarrow[-C_{19}H_9N_2]{465°C - 655°C} Cd$$

$$[C_{25}H_{23}N_3O_4Al]^+Cl^- \xrightarrow[-H_2O,\ HCl,\ NO_2]{90°C - 200°C} [C_{25}H_{20}N_2OAl] \xrightarrow[-C_3H_5NO]{200°C - 325°C} [C_{22}H_{15}NAl] \xrightarrow[-C_9H_6N]{325°C - 400°C}$$

$$[C_{13}H_9Al] \xrightarrow[-C_4H_4]{400°C - 490°C} [C_9H_5Al] \xrightarrow[-C_9H_5]{490°C - 710°C} Al$$

$$[C_{25}H_{21}ClN_3O_3La]H_2O \xrightarrow[-H_2O]{35°C - 200°C} [C_{25}H_{21}ClN_3O_3La] \xrightarrow[-C_5H_3NOCl]{200°C - 265°C} [C_{20}H_{18}N_2O_2La] \xrightarrow[-C_4H_4]{265°C - 320°C}$$

$$[C_{16}H_{14}N_2O_2La] \xrightarrow[-N_2O]{320°C - 360°C} [C_{16}H_{14}OLa] \xrightarrow[-CH_2O]{360°C - 385°C} [C_{15}H_{12}La] \xrightarrow[-C_{15}H_{12}]{385°C - 625°C} La$$

Scheme S1: Thermo gravimetric degradation steps for the prepared Mixed salen/8-hydroxy quinoline complexes with Ni(II), Cd(II), Al(III), La(III) from ambient temperature to 800 °C at heating rate of 10 °C / min

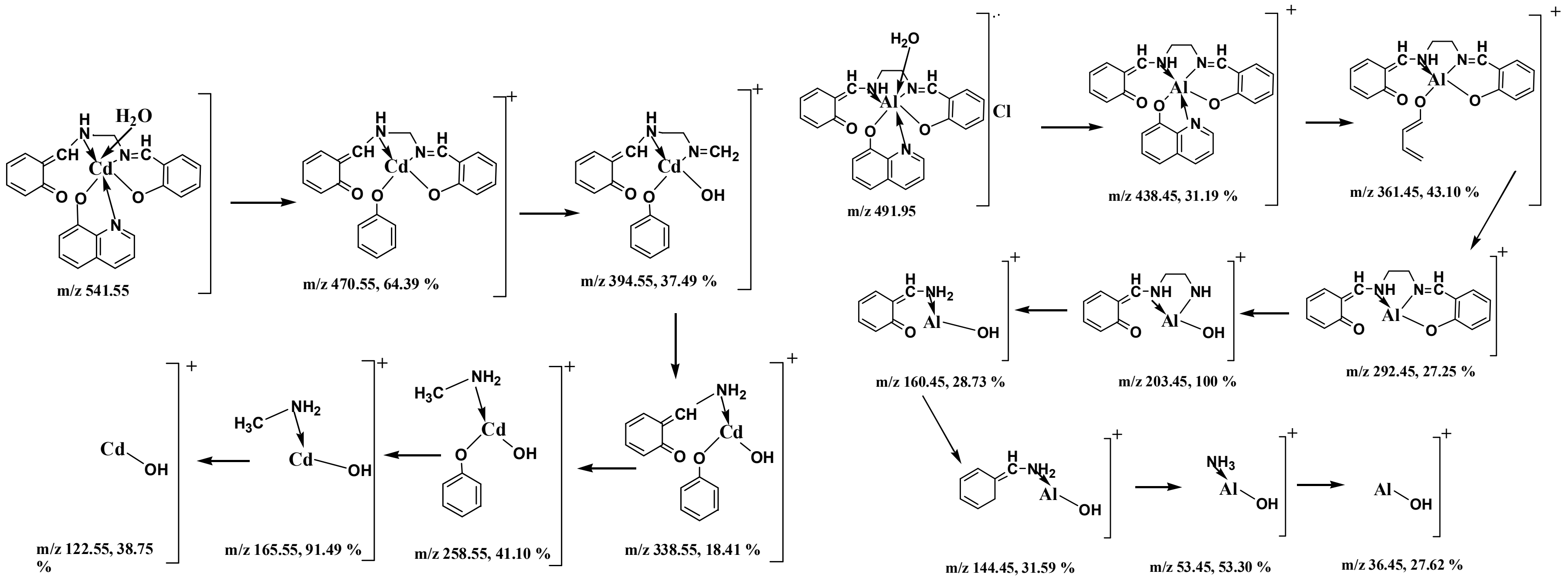

Scheme S2: Mass fragmentation of CdSQ and AlSQ complexes
